# Supplementary material for: Pyruvate Dehydrogenase Kinase 1 inhibition mediated oxidative phosphorylation enhancement in cartilage promotes osteoarthritis progression
Source: BMC Musculoskelet Disord. 2023 Jul 20;24:597. doi: 10.1186/s12891-023-06585-6 (PMC10357736; doi:10.1186/s12891-023-06585-6)
Supplement: Supplementary file 1 — Additional file 1. [file 12891_2023_6585_MOESM1_ESM.zip › MF.Gsea.1653627316242/GOMF_HELICASE_ACTIVITY.html]

Details for gene set GOMF\_HELICASE\_ACTIVITY[GSEA]

|  || Dataset | 55457\_collapsed\_to\_symbols.cls.cls #OA\_versus\_normal.cls.cls #OA\_versus\_normal\_repos |
| Phenotype | cls.cls#OA\_versus\_normal\_repos |
| Upregulated in class | normal |
| GeneSet | GOMF\_HELICASE\_ACTIVITY |
| Enrichment Score (ES) | -0.4307649 |
| Normalized Enrichment Score (NES) | -1.9367373 |
| Nominal p-value | 0.0 |
| FDR q-value | 0.034074683 |
| FWER p-Value | 0.146 |
Table: GSEA Results Summary

  

Fig 1: Enrichment plot: GOMF\_HELICASE\_ACTIVITY      
 Profile of the Running ES Score & Positions of GeneSet Members on the Rank Ordered List

  

| SYMBOL | TITLE | RANK IN GENE LIST | RANK METRIC SCORE | RUNNING ES | CORE ENRICHMENT || 1 | MCM5 | minichromosome maintenance complex component 5 [Source:HGNC Symbol;Acc:HGNC:6948] | 351 | 0.724 | -0.0118 | No |
| 2 | RFC3 | replication factor C subunit 3 [Source:HGNC Symbol;Acc:HGNC:9971] | 570 | 0.633 | -0.0152 | No |
| 3 | MCM2 | minichromosome maintenance complex component 2 [Source:HGNC Symbol;Acc:HGNC:6944] | 888 | 0.546 | -0.0282 | No |
| 4 | RFC4 | replication factor C subunit 4 [Source:HGNC Symbol;Acc:HGNC:9972] | 897 | 0.543 | -0.0171 | No |
| 5 | DDX23 | DEAD-box helicase 23 [Source:HGNC Symbol;Acc:HGNC:17347] | 913 | 0.538 | -0.0066 | No |
| 6 | DHX29 | DExH-box helicase 29 [Source:HGNC Symbol;Acc:HGNC:15815] | 1012 | 0.515 | -0.0032 | No |
| 7 | XRCC5 | X-ray repair cross complementing 5 [Source:HGNC Symbol;Acc:HGNC:12833] | 1018 | 0.513 | 0.0075 | No |
| 8 | MCM6 | minichromosome maintenance complex component 6 [Source:HGNC Symbol;Acc:HGNC:6949] | 1131 | 0.490 | 0.0093 | No |
| 9 | RFC2 | replication factor C subunit 2 [Source:HGNC Symbol;Acc:HGNC:9970] | 1247 | 0.467 | 0.0104 | No |
| 10 | CHD1L | chromodomain helicase DNA binding protein 1 like [Source:HGNC Symbol;Acc:HGNC:1916] | 1320 | 0.454 | 0.0146 | No |
| 11 | DDX60 | DExD/H-box helicase 60 [Source:HGNC Symbol;Acc:HGNC:25942] | 1385 | 0.445 | 0.0192 | No |
| 12 | RAD51 | RAD51 recombinase [Source:HGNC Symbol;Acc:HGNC:9817] | 1581 | 0.416 | 0.0130 | No |
| 13 | HLTF | helicase like transcription factor [Source:HGNC Symbol;Acc:HGNC:11099] | 1620 | 0.411 | 0.0189 | No |
| 14 | DDX6 | DEAD-box helicase 6 [Source:HGNC Symbol;Acc:HGNC:2747] | 1687 | 0.401 | 0.0224 | No |
| 15 | MTREX | Mtr4 exosome RNA helicase [Source:HGNC Symbol;Acc:HGNC:18734] | 2138 | 0.346 | -0.0054 | No |
| 16 | IFIH1 | interferon induced with helicase C domain 1 [Source:HGNC Symbol;Acc:HGNC:18873] | 2204 | 0.338 | -0.0032 | No |
| 17 | DHX57 | DExH-box helicase 57 [Source:HGNC Symbol;Acc:HGNC:20086] | 2262 | 0.331 | -0.0005 | No |
| 18 | DSCC1 | DNA replication and sister chromatid cohesion 1 [Source:HGNC Symbol;Acc:HGNC:24453] | 2362 | 0.319 | -0.0013 | No |
| 19 | RUVBL2 | RuvB like AAA ATPase 2 [Source:HGNC Symbol;Acc:HGNC:10475] | 2778 | 0.272 | -0.0279 | No |
| 20 | DDX4 | DEAD-box helicase 4 [Source:HGNC Symbol;Acc:HGNC:18700] | 2788 | 0.271 | -0.0228 | No |
| 21 | DDX50 | DExD-box helicase 50 [Source:HGNC Symbol;Acc:HGNC:17906] | 3167 | 0.234 | -0.0473 | No |
| 22 | SMARCA2 | "SWI/SNF related, matrix associated, actin dependent regulator of chromatin, subfamily a, member 2 [Source:HGNC Symbol;Acc:HGNC:11098]" | 3281 | 0.222 | -0.0513 | No |
| 23 | DDX1 | DEAD-box helicase 1 [Source:HGNC Symbol;Acc:HGNC:2734] | 3305 | 0.221 | -0.0484 | No |
| 24 | ERCC2 | "ERCC excision repair 2, TFIIH core complex helicase subunit [Source:HGNC Symbol;Acc:HGNC:3434]" | 3544 | 0.198 | -0.0627 | No |
| 25 | MCM3 | minichromosome maintenance complex component 3 [Source:HGNC Symbol;Acc:HGNC:6945] | 3675 | 0.188 | -0.0688 | No |
| 26 | DHX58 | DExH-box helicase 58 [Source:HGNC Symbol;Acc:HGNC:29517] | 4130 | 0.143 | -0.1013 | No |
| 27 | POLQ | DNA polymerase theta [Source:HGNC Symbol;Acc:HGNC:9186] | 4293 | 0.129 | -0.1112 | No |
| 28 | DHX40 | DEAH-box helicase 40 [Source:HGNC Symbol;Acc:HGNC:18018] | 4358 | 0.124 | -0.1135 | No |
| 29 | UPF1 | UPF1 RNA helicase and ATPase [Source:HGNC Symbol;Acc:HGNC:9962] | 4434 | 0.118 | -0.1168 | No |
| 30 | DDX19B | DEAD-box helicase 19B [Source:HGNC Symbol;Acc:HGNC:2742] | 4500 | 0.113 | -0.1195 | No |
| 31 | CHD9 | chromodomain helicase DNA binding protein 9 [Source:HGNC Symbol;Acc:HGNC:25701] | 4520 | 0.112 | -0.1185 | No |
| 32 | DDX28 | DEAD-box helicase 28 [Source:HGNC Symbol;Acc:HGNC:17330] | 4602 | 0.105 | -0.1226 | No |
| 33 | ASCC3 | activating signal cointegrator 1 complex subunit 3 [Source:HGNC Symbol;Acc:HGNC:18697] | 4689 | 0.097 | -0.1273 | No |
| 34 | XRCC6 | X-ray repair cross complementing 6 [Source:HGNC Symbol;Acc:HGNC:4055] | 4699 | 0.096 | -0.1259 | No |
| 35 | DDX47 | DEAD-box helicase 47 [Source:HGNC Symbol;Acc:HGNC:18682] | 4789 | 0.088 | -0.1309 | No |
| 36 | BLM | BLM RecQ like helicase [Source:HGNC Symbol;Acc:HGNC:1058] | 4843 | 0.084 | -0.1333 | No |
| 37 | DDX41 | DEAD-box helicase 41 [Source:HGNC Symbol;Acc:HGNC:18674] | 5004 | 0.073 | -0.1442 | No |
| 38 | DHX8 | DEAH-box helicase 8 [Source:HGNC Symbol;Acc:HGNC:2749] | 5531 | 0.030 | -0.1848 | No |
| 39 | SMARCA1 | "SWI/SNF related, matrix associated, actin dependent regulator of chromatin, subfamily a, member 1 [Source:HGNC Symbol;Acc:HGNC:11097]" | 5723 | 0.014 | -0.1994 | No |
| 40 | DDX46 | DEAD-box helicase 46 [Source:HGNC Symbol;Acc:HGNC:18681] | 5744 | 0.012 | -0.2007 | No |
| 41 | RAD54B | RAD54 homolog B [Source:HGNC Symbol;Acc:HGNC:17228] | 5753 | 0.012 | -0.2011 | No |
| 42 | BRIP1 | BRCA1 interacting helicase 1 [Source:HGNC Symbol;Acc:HGNC:20473] | 5885 | 0.003 | -0.2113 | No |
| 43 | MCM4 | minichromosome maintenance complex component 4 [Source:HGNC Symbol;Acc:HGNC:6947] | 6003 | -0.005 | -0.2203 | No |
| 44 | RAD50 | RAD50 double strand break repair protein [Source:HGNC Symbol;Acc:HGNC:9816] | 6474 | -0.040 | -0.2563 | No |
| 45 | DDX58 | DExD/H-box helicase 58 [Source:HGNC Symbol;Acc:HGNC:19102] | 6480 | -0.041 | -0.2558 | No |
| 46 | HELLS | "helicase, lymphoid specific [Source:HGNC Symbol;Acc:HGNC:4861]" | 6534 | -0.045 | -0.2589 | No |
| 47 | SNRNP200 | small nuclear ribonucleoprotein U5 subunit 200 [Source:HGNC Symbol;Acc:HGNC:30859] | 7249 | -0.099 | -0.3127 | No |
| 48 | DDX42 | DEAD-box helicase 42 [Source:HGNC Symbol;Acc:HGNC:18676] | 7447 | -0.114 | -0.3256 | No |
| 49 | RUVBL1 | RuvB like AAA ATPase 1 [Source:HGNC Symbol;Acc:HGNC:10474] | 7477 | -0.116 | -0.3254 | No |
| 50 | ATRX | ATRX chromatin remodeler [Source:HGNC Symbol;Acc:HGNC:886] | 7551 | -0.123 | -0.3284 | No |
| 51 | EIF4A3 | eukaryotic translation initiation factor 4A3 [Source:HGNC Symbol;Acc:HGNC:18683] | 7646 | -0.130 | -0.3330 | No |
| 52 | AQR | aquarius intron-binding spliceosomal factor [Source:HGNC Symbol;Acc:HGNC:29513] | 7654 | -0.131 | -0.3307 | No |
| 53 | SMARCAL1 | "SWI/SNF related, matrix associated, actin dependent regulator of chromatin, subfamily a like 1 [Source:HGNC Symbol;Acc:HGNC:11102]" | 7686 | -0.133 | -0.3302 | No |
| 54 | TTF2 | transcription termination factor 2 [Source:HGNC Symbol;Acc:HGNC:12398] | 7764 | -0.139 | -0.3333 | No |
| 55 | RAD54L2 | RAD54 like 2 [Source:HGNC Symbol;Acc:HGNC:29123] | 7872 | -0.146 | -0.3385 | No |
| 56 | ERCC6 | "ERCC excision repair 6, chromatin remodeling factor [Source:HGNC Symbol;Acc:HGNC:3438]" | 7939 | -0.150 | -0.3404 | No |
| 57 | DDX49 | DEAD-box helicase 49 [Source:HGNC Symbol;Acc:HGNC:18684] | 7945 | -0.151 | -0.3375 | No |
| 58 | DDX18 | DEAD-box helicase 18 [Source:HGNC Symbol;Acc:HGNC:2741] | 7996 | -0.154 | -0.3381 | No |
| 59 | DHX30 | DExH-box helicase 30 [Source:HGNC Symbol;Acc:HGNC:16716] | 8111 | -0.164 | -0.3435 | No |
| 60 | SKIV2L | Ski2 like RNA helicase [Source:HGNC Symbol;Acc:HGNC:10898] | 8395 | -0.185 | -0.3616 | No |
| 61 | SETX | senataxin [Source:HGNC Symbol;Acc:HGNC:445] | 8398 | -0.185 | -0.3578 | No |
| 62 | ERCC3 | "ERCC excision repair 3, TFIIH core complex helicase subunit [Source:HGNC Symbol;Acc:HGNC:3435]" | 8669 | -0.206 | -0.3745 | No |
| 63 | MCM9 | minichromosome maintenance 9 homologous recombination repair factor [Source:HGNC Symbol;Acc:HGNC:21484] | 8781 | -0.215 | -0.3785 | No |
| 64 | DDX25 | DEAD-box helicase 25 [Source:HGNC Symbol;Acc:HGNC:18698] | 8815 | -0.218 | -0.3764 | No |
| 65 | CHD4 | chromodomain helicase DNA binding protein 4 [Source:HGNC Symbol;Acc:HGNC:1919] | 8917 | -0.226 | -0.3794 | No |
| 66 | EIF4A2 | eukaryotic translation initiation factor 4A2 [Source:HGNC Symbol;Acc:HGNC:3284] | 9251 | -0.252 | -0.4000 | No |
| 67 | EP400 | E1A binding protein p400 [Source:HGNC Symbol;Acc:HGNC:11958] | 9304 | -0.256 | -0.3986 | No |
| 68 | RFC5 | replication factor C subunit 5 [Source:HGNC Symbol;Acc:HGNC:9973] | 9499 | -0.274 | -0.4078 | No |
| 69 | DDX24 | DEAD-box helicase 24 [Source:HGNC Symbol;Acc:HGNC:13266] | 9588 | -0.282 | -0.4086 | No |
| 70 | YTHDC2 | YTH domain containing 2 [Source:HGNC Symbol;Acc:HGNC:24721] | 9872 | -0.309 | -0.4241 | Yes |
| 71 | GTF2F2 | general transcription factor IIF subunit 2 [Source:HGNC Symbol;Acc:HGNC:4653] | 9876 | -0.309 | -0.4176 | Yes |
| 72 | HELZ | helicase with zinc finger [Source:HGNC Symbol;Acc:HGNC:16878] | 9889 | -0.310 | -0.4119 | Yes |
| 73 | SMARCA4 | "SWI/SNF related, matrix associated, actin dependent regulator of chromatin, subfamily a, member 4 [Source:HGNC Symbol;Acc:HGNC:11100]" | 10009 | -0.322 | -0.4142 | Yes |
| 74 | WRN | WRN RecQ like helicase [Source:HGNC Symbol;Acc:HGNC:12791] | 10010 | -0.323 | -0.4072 | Yes |
| 75 | MCM7 | minichromosome maintenance complex component 7 [Source:HGNC Symbol;Acc:HGNC:6950] | 10032 | -0.325 | -0.4019 | Yes |
| 76 | RECQL | RecQ like helicase [Source:HGNC Symbol;Acc:HGNC:9948] | 10124 | -0.333 | -0.4018 | Yes |
| 77 | DHX32 | DEAH-box helicase 32 (putative) [Source:HGNC Symbol;Acc:HGNC:16717] | 10127 | -0.334 | -0.3947 | Yes |
| 78 | DDX51 | DEAD-box helicase 51 [Source:HGNC Symbol;Acc:HGNC:20082] | 10133 | -0.334 | -0.3879 | Yes |
| 79 | RECQL5 | RecQ like helicase 5 [Source:HGNC Symbol;Acc:HGNC:9950] | 10144 | -0.335 | -0.3814 | Yes |
| 80 | DHX35 | DEAH-box helicase 35 [Source:HGNC Symbol;Acc:HGNC:15861] | 10215 | -0.342 | -0.3795 | Yes |
| 81 | IGHMBP2 | immunoglobulin mu DNA binding protein 2 [Source:HGNC Symbol;Acc:HGNC:5542] | 10248 | -0.346 | -0.3745 | Yes |
| 82 | CHD3 | chromodomain helicase DNA binding protein 3 [Source:HGNC Symbol;Acc:HGNC:1918] | 10269 | -0.347 | -0.3686 | Yes |
| 83 | CHD2 | chromodomain helicase DNA binding protein 2 [Source:HGNC Symbol;Acc:HGNC:1917] | 10467 | -0.370 | -0.3760 | Yes |
| 84 | DHX15 | DEAH-box helicase 15 [Source:HGNC Symbol;Acc:HGNC:2738] | 10498 | -0.372 | -0.3703 | Yes |
| 85 | DHX9 | DExH-box helicase 9 [Source:HGNC Symbol;Acc:HGNC:2750] | 10627 | -0.386 | -0.3720 | Yes |
| 86 | WRNIP1 | WRN helicase interacting protein 1 [Source:HGNC Symbol;Acc:HGNC:20876] | 10909 | -0.418 | -0.3849 | Yes |
| 87 | DDX10 | DEAD-box helicase 10 [Source:HGNC Symbol;Acc:HGNC:2735] | 11029 | -0.434 | -0.3849 | Yes |
| 88 | G3BP1 | G3BP stress granule assembly factor 1 [Source:HGNC Symbol;Acc:HGNC:30292] | 11117 | -0.445 | -0.3821 | Yes |
| 89 | DDX21 | DExD-box helicase 21 [Source:HGNC Symbol;Acc:HGNC:2744] | 11120 | -0.446 | -0.3726 | Yes |
| 90 | TDRD12 | tudor domain containing 12 [Source:HGNC Symbol;Acc:HGNC:25044] | 11283 | -0.469 | -0.3751 | Yes |
| 91 | DDX39A | DExD-box helicase 39A [Source:HGNC Symbol;Acc:HGNC:17821] | 11359 | -0.480 | -0.3706 | Yes |
| 92 | DDX56 | DEAD-box helicase 56 [Source:HGNC Symbol;Acc:HGNC:18193] | 11386 | -0.484 | -0.3622 | Yes |
| 93 | MRE11 | "MRE11 homolog, double strand break repair nuclease [Source:HGNC Symbol;Acc:HGNC:7230]" | 11458 | -0.497 | -0.3570 | Yes |
| 94 | BTAF1 | B-TFIID TATA-box binding protein associated factor 1 [Source:HGNC Symbol;Acc:HGNC:17307] | 11480 | -0.499 | -0.3479 | Yes |
| 95 | DDX52 | DExD-box helicase 52 [Source:HGNC Symbol;Acc:HGNC:20038] | 11484 | -0.499 | -0.3373 | Yes |
| 96 | NAV2 | neuron navigator 2 [Source:HGNC Symbol;Acc:HGNC:15997] | 11619 | -0.520 | -0.3366 | Yes |
| 97 | ERCC6L | "ERCC excision repair 6 like, spindle assembly checkpoint helicase [Source:HGNC Symbol;Acc:HGNC:20794]" | 11663 | -0.527 | -0.3285 | Yes |
| 98 | DDX17 | DEAD-box helicase 17 [Source:HGNC Symbol;Acc:HGNC:2740] | 11716 | -0.535 | -0.3210 | Yes |
| 99 | CHD5 | chromodomain helicase DNA binding protein 5 [Source:HGNC Symbol;Acc:HGNC:16816] | 11776 | -0.545 | -0.3139 | Yes |
| 100 | DDX19A | DEAD-box helicase 19A [Source:HGNC Symbol;Acc:HGNC:25628] | 11821 | -0.553 | -0.3054 | Yes |
| 101 | CHD8 | chromodomain helicase DNA binding protein 8 [Source:HGNC Symbol;Acc:HGNC:20153] | 11825 | -0.554 | -0.2936 | Yes |
| 102 | SMARCA5 | "SWI/SNF related, matrix associated, actin dependent regulator of chromatin, subfamily a, member 5 [Source:HGNC Symbol;Acc:HGNC:11101]" | 11838 | -0.557 | -0.2825 | Yes |
| 103 | DHX38 | DEAH-box helicase 38 [Source:HGNC Symbol;Acc:HGNC:17211] | 11860 | -0.562 | -0.2720 | Yes |
| 104 | RECQL4 | RecQ like helicase 4 [Source:HGNC Symbol;Acc:HGNC:9949] | 11927 | -0.578 | -0.2647 | Yes |
| 105 | RTEL1 | regulator of telomere elongation helicase 1 [Source:HGNC Symbol;Acc:HGNC:15888] | 11968 | -0.587 | -0.2551 | Yes |
| 106 | DDX54 | DEAD-box helicase 54 [Source:HGNC Symbol;Acc:HGNC:20084] | 11969 | -0.587 | -0.2424 | Yes |
| 107 | DHX34 | DExH-box helicase 34 [Source:HGNC Symbol;Acc:HGNC:16719] | 12040 | -0.603 | -0.2349 | Yes |
| 108 | TWNK | twinkle mtDNA helicase [Source:HGNC Symbol;Acc:HGNC:1160] | 12069 | -0.612 | -0.2238 | Yes |
| 109 | SUPV3L1 | Suv3 like RNA helicase [Source:HGNC Symbol;Acc:HGNC:11471] | 12087 | -0.616 | -0.2119 | Yes |
| 110 | EIF4A1 | eukaryotic translation initiation factor 4A1 [Source:HGNC Symbol;Acc:HGNC:3282] | 12111 | -0.623 | -0.2002 | Yes |
| 111 | DNA2 | DNA replication helicase/nuclease 2 [Source:HGNC Symbol;Acc:HGNC:2939] | 12148 | -0.631 | -0.1894 | Yes |
| 112 | RAD54L | RAD54 like [Source:HGNC Symbol;Acc:HGNC:9826] | 12204 | -0.646 | -0.1797 | Yes |
| 113 | DICER1 | "dicer 1, ribonuclease III [Source:HGNC Symbol;Acc:HGNC:17098]" | 12215 | -0.650 | -0.1665 | Yes |
| 114 | DDX31 | DEAD-box helicase 31 [Source:HGNC Symbol;Acc:HGNC:16715] | 12288 | -0.672 | -0.1576 | Yes |
| 115 | DDX27 | DEAD-box helicase 27 [Source:HGNC Symbol;Acc:HGNC:15837] | 12359 | -0.695 | -0.1480 | Yes |
| 116 | DHX16 | DEAH-box helicase 16 [Source:HGNC Symbol;Acc:HGNC:2739] | 12414 | -0.712 | -0.1369 | Yes |
| 117 | SRCAP | Snf2 related CREBBP activator protein [Source:HGNC Symbol;Acc:HGNC:16974] | 12542 | -0.769 | -0.1302 | Yes |
| 118 | CHD7 | chromodomain helicase DNA binding protein 7 [Source:HGNC Symbol;Acc:HGNC:20626] | 12591 | -0.791 | -0.1168 | Yes |
| 119 | DDX11 | DEAD/H-box helicase 11 [Source:HGNC Symbol;Acc:HGNC:2736] | 12607 | -0.803 | -0.1006 | Yes |
| 120 | DDX3Y | DEAD-box helicase 3 Y-linked [Source:HGNC Symbol;Acc:HGNC:2699] | 12728 | -0.890 | -0.0908 | Yes |
| 121 | CHD1 | chromodomain helicase DNA binding protein 1 [Source:HGNC Symbol;Acc:HGNC:1915] | 12745 | -0.908 | -0.0724 | Yes |
| 122 | DDX39B | DExD-box helicase 39B [Source:HGNC Symbol;Acc:HGNC:13917] | 12783 | -0.941 | -0.0550 | Yes |
| 123 | DDX3X | DEAD-box helicase 3 X-linked [Source:HGNC Symbol;Acc:HGNC:2745] | 12788 | -0.949 | -0.0348 | Yes |
| 124 | DDX43 | DEAD-box helicase 43 [Source:HGNC Symbol;Acc:HGNC:18677] | 12800 | -0.969 | -0.0147 | Yes |
| 125 | DDX5 | DEAD-box helicase 5 [Source:HGNC Symbol;Acc:HGNC:2746] | 12834 | -1.038 | 0.0052 | Yes |
Table: GSEA details [plain text format]

  

Fig 2: GOMF\_HELICASE\_ACTIVITY      
 Blue-Pink O' Gram in the Space of the Analyzed GeneSet

  

Fig 3: GOMF\_HELICASE\_ACTIVITY: Random ES distribution      
 Gene set null distribution of ES for **GOMF\_HELICASE\_ACTIVITY**
